# Activated gliosis, accumulation of amyloid β, and hyperphosphorylation of tau in aging canines with and without cognitive decline

**DOI:** 10.3389/fnagi.2023.1128521

**Published:** 2023-04-27

**Authors:** Amelia D. Hines, Stephanie McGrath, Amanda S. Latham, Breonna Kusick, Lisa Mulligan, McKenzie L. Richards, Julie A. Moreno

**Affiliations:** ^1^Department of Environmental and Radiological Health Sciences, College of Veterinary Medicine and Biomedical Sciences, Colorado State University, Fort Collins, CO, United States; ^2^Department of Clinical Sciences, College of Veterinary Medicine and Biomedical Sciences, Colorado State University, Fort Collins, CO, United States

**Keywords:** aging, canines, neuroinflammation, tau hyperphosphorylation, amyloid beta (1–42)

## Abstract

Canine cognitive dysfunction (CCD) syndrome is a well-recognized naturally occurring disease in aged dogs, with a remarkably similar disease course, both in its clinical presentation and neuropathological changes, as humans with Alzheimer’s disease (AD). Similar to human AD patients this naturally occurring disease is found in the aging canine population however, there is little understanding of how the canine brain ages pathologically. It is well known that in neurodegenerative diseases, there is an increase in inflamed glial cells as well as an accumulation of hyperphosphorylation of tau (P-tau) and amyloid beta (Aβ_1-42_). These pathologies increase neurotoxic signaling and eventual neuronal loss. We assessed these brain pathologies in aged canines and found an increase in the number of glial cells, both astrocytes and microglia, and the activation of astrocytes indicative of neuroinflammation. A rise in the aggregated protein Aβ_1-42_ and hyperphosphorylated tau, at Threonine 181 and 217, in the cortical brain regions of aging canines. We then asked if any of these aged canines had CCD utilizing the only current diagnostic, owner questionnaires, verifying positive or severe CCD had pathologies of gliosis and accumulation of Aβ_1-42_ like their aged, matched controls. However uniquely the CCD dogs had P-tau at T217. Therefore, this phosphorylation site of tau at threonine 217 may be a predictor for CCD.

## 1. Introduction

Age is the number one risk factor for cognitive decline in canines and humans; however, the etiology is not known. Studying aging dogs with and without canine cognitive dysfunction (CCD) syndrome shows promise for a better understanding of human age-related neurodegeneration. Canine cognitive dysfunction is considered an age-related disease, with the prevalence ranging from 14–35% of the senior canine population, exponentially increasing with age (Neilson et al., 2001; Azkona et al., 2009; Salvin et al., 2010). In a two-year longitudinal study of 51 dogs over the age of 8 years, 33% of dogs with normal cognitive status progressed to mild cognitive impairment and 22% of dogs with mild cognitive impairment progressed to CCD (Schutt et al., 2015). In another study of 180 geriatric dogs (aged 11–16 years), 28% of dogs 11–12 years old and 68% of dogs 15–16 years old exhibited signs of cognitive impairment (Neilson et al., 2001). Unlike commonly used transgenic, canines age and develop CCD naturally, making them an ideal avenue for studying brain aging and disease. With the aging canine population steadily increasing, it is essential to study these aging dogs, especially given the implications and translational value for their human counterpart. Therefore, a clearer understanding of the neuropathogenesis of aging canine brains is essential for the future development of diagnostic tests and to facilitate timely intervention, for human patients and canines alike.

Although research has shown neuropathology within the aged canine brain, information on CCD-specific pathology, outside of those associated with age, is needed. The aging canine brain, similar to humans, correlates with declined cognition and neuronal pathology (Cummings and Cotman, 1995; Head et al., 1998; Rofina et al., 2006; Mormino and Papp, 2018). One common pathology in brain aging is an increase in glial inflammation, which usually affects the cortex and hippocampus (Beach et al., 1989). Although glia, especially microglia and astrocytes, are critical for normal brain function, neuroinflammation in the central nervous system (CNS) induces reactive cell phenotypes. Microglial proliferation and reactivity play an important role in neurodegenerative responses. Studies show that aged canines exhibit greater levels of the microglial marker ionized calcium binding adaptor molecule 1 (Iba1) in the hippocampus (Hwang et al., 2008; Ozawa et al., 2016; Smolek et al., 2016). Microglia are also critical mediators of astrocyte activation through their release of proinflammatory chemokines and cytokines (Liddelow et al., 2017). Like microglia, activated, or A1, astrocytes secrete pro-inflammatory molecules and promote neuronal death over time. They are also a source of complement system proteins, especially C3, which mediates neuronal damage in models of AD (Wu et al., 2019; Pekna and Pekny, 2021).

In addition to glial inflammation, the aggregation and deposition of amyloid-β_1-42_ (Aβ_1-42_) as insoluble, extracellular plaques is a common brain pathology seen in humans with neurodegenerative disease, especially in the cerebral cortex. Aβ_1-42_ is also present in canines with cognitive decline, and cognitive impairment in aged canines has been strongly associated with the accumulation of Aβ_1-42_ (Pugliese et al., 2005; Rofina et al., 2006; Barnes et al., 2016). The amino acid sequence of β-amyloid is identical between humans and dogs (Johnstone et al., 1991) allowing for the use of similar reagents across species, further establishing the canine as an appropriate model. In addition to Aβ_1-42,_ hyperphosphorylation of the tau protein (P-tau) is also commonly found in humans with AD and has also been recently discovered in CCD dogs (Abey et al., 2021). Thus, we investigated the occurrence of P-tau and Aβ_1-42_ in our aged canine brains, using antibodies for multiple phosphorylation sites of tau and techniques to verify the role of these misfolded proteins in the aging canine.

## 2. Materials and methods

### 2.1. Sample collection and preparation

Forty-seven canine brains were obtained from privately owned dogs by owner consent after canines were euthanized and necropsied. The brains were collected at the James L. Voss Veterinary Teaching Hospital at Colorado State University. Canine brains were fixed in 10% neutral buffer formalin for a minimum of 72 h. After fixation, brains were processed using a tissue processor (Leica TP 1020) and embedded in paraffin wax using a tissue embedder (Leica EG 1160). Tissue blocks containing the cortex were cut from the frontal lobe when possible, however due to the condition of brain tissue, this was not always possible. Hippocampus blocks were selected by finding the most intact area of hippocampus available. Using a microtome (ThermoFisher HM1030), sections were cut at 5 μm and mounted on a charged slide. The details for euthanasia, age, and weights of the canines are listed in Table 1.

**Table 1:** Aged canine cause of death, age and weight information.

Canine #	Age (years)	Weight (kg)	Reason for Euthanasia
Ca110	2.2 years	N/A	N/A
Ca111	1.2 years	14.6	N/A
Ca112	4.7 years	25.6	N/A
Ca113	4.2 years	N/A	N/A
Ca152	15 years	44	Severe OA, inability to rise
Ca153	9 years	7.8	Nasal carcinoma
Ca154	8 years	39.4	Hemiangiosarcoma
Ca155	12 years	7.23	Severe lethargy, mental obdundation
Ca156	11 months	5.79	GME
Ca159	15 years	21.1	Nasal carcinoma with multifocal metastisis
Ca160	8 years	8.41	Hind limb paralysis
Ca169	8 years	30	Cardiac mass
Ca170	9 years	18.7	Severe cushing’s disease
Ca171	13 years	26.8	Disseminated lymphoma (stage V)
Ca172	10 years	26.1	Left temporal osteosarcoma
Ca173	14 years	8	DOA – cardiac arrest
Ca174	11 years	19.6	Multifocal carcinomas and sarcomas
Ca175	13.5 years	N/A	N/A
Ca177	16 years	N/A	N/A
Ca178	15 years	N/A	N/A
Ca183	14 years	11.3	Decline, unable to walk, possible bladder stones
Ca186	9 years	N/A	N/A
Ca187	13yo	N/A	N/A
Ca188	9 years	54	Pericardial effusion, peritoneal effusion, acute lethargy and anorexia
Ca189	11.5 years	43.3	Metastatic mast cell tumor
Ca192	10 years	N/A	Hemoabdomen
Ca193	8 years	15.7	Severe nasopharyngeal obstruction with fibrous scar tissue
Ca194	10 years	10.1	Labored breathing
Ca195	12.5 years	23.6	DCM due to grain-free diet
Ca196	12 years	20	Abdominal mass, difficulty defecating and urinating
Ca197	12 years	30	Bicavity effusion
Ca198	13.5 years	35	Anorexia, lethargy, obtunded on presentation, abdominal effusion, hypovolemic, diffuse hepatic nodules
Ca199	13 years	8.4	Decline due to age
Ca256	8 years	20	Hemangiosarcoma
Ca264	13 years	9.7	Respiratory distress
Ca289	15 years	4.9	Chronic cough, hepatomealgy, degenerative heart disease
Ca351	13.6 years	N/A	Degenerative mitral disease, bilateral adrenomegaly, hypoadrenocorticism
Ca800	12.4 years	N/A	Severe emaciation, general decompensation

N/A = data not available.

### 2.2. Immunohistochemical staining and microscopy imaging analysis

Immunohistochemistry was used to visualize a variety of neurological antibodies including: GFAP (DAKO Cat. #20334), Iba1 (Abcam 5,076), S100β (Abcam 41,428), AT270 (Thermo Fisher MN1050), T217 (Abclonal AP1233) and Aβ_1-42_ (Thermo Fisher 44344). Sections were dewaxed in xylene and rehydrated through graded ethanol’s, before undergoing antigen retrieval in 0.01 M sodium citrate for 20 min at 95°C. Endoperoxides were removed by incubating sections in 0.30% hydrogen peroxide and water solution for 30 min. Blocking of non-specific labeling was performed with 10% hose serum in Tris-A in 2% BSA [2% bovine serum albumin (BSA) and 2% Triton-X in tris buffered saline (TBS)]. Sections were incubated overnight at 4°C in primary antibodies diluted with Tris-A in 2% BSA [GFAP 1:400, Iba1 1:400, Aβ_1-42_ 1:200, S100β 1:750, T217 1:100, T181 1:500]. Sections were then washed with Tris-A in 2% BSA and incubated for an hour against their corresponding biotinylated secondary antibodies, goat anti-rabbit or horse anti-mouse (Vector labs), diluted in 10% horse serum in Tris-A 2% BSA 1:250. Slides were counterstained with hematoxylin, rinsed in bluing reagent and dehydrated through graded ethanol followed by xylene, mounted with mounting media and cover slipped with #1 cover glass.

Slides were visualized using Olympus BX51 microscope and Olympus DP70 camera and saved as virtual slide images at 20X objective. Quantitative analysis was performed using Olympus CellSens Dimension Desktop 3.1 using Count and Measure quantification application. After scanning the tissue on the slide, a representative area was selected for quantification by cell counting using CellSens software. The representative area was chosen after looking at the full area of cortex or hippocampus. The size of the area chosen was calculated in μm^2^, and the number of positive cells in that area were then normalized over an area of 1 mm^2^.

### 2.3. Immunofluorescent staining and microscopy imaging analysis

Immunofluorescence was used to visualize co-expression of certain proteins in glial or neuronal cell types: C3(Abcam 181,147) [1:250] and S100β (Abcam 41,428) [1:750]. Slides were visualized using BX63 fluorescence microscope equipped with a Hamamatsu ORCA-flash 4.0 LT CCD camera and collected using Olympus CellSens software version 3.1. Images were taken as virtual slide images at 10X objective to be analyzed on Olympus Cell Sense technology using Count and Measure quantification application.

### 2.4. Analysis of canine owner questionnaires for canine cognitive decline

Two commonly used veterinary scales for CCD were given to client owners following the necropsy of their canine, Canine Cognitive Dysfunction Rating Scale (CCDR) (Salvin et al., 2011a) and the Canine Dementia Scale (CADES) (Madari et al., 2015). These questionnaires were either collected in person by veterinary staff or distributed online, when in person communication was not an option.

### 2.5. Statistical analysis

All data is presented as mean ± SEM. Differences between experimental groups were analyzed using an unpaired *t*-test using Prism GraphPad. Significance is denoted as * = *p* ≤ 0.05.

## 3. Results

### 3.1. Increased glial inflammation in the cortex and hippocampus of aged canines

Young (1–4 years of age) and aged (over 8 years of age) canine brains were stained for glial markers and quantified in two brain regions related to neurodegenerative disease, the cortex and hippocampus. Canines between the ages of 5–7 years of age were excluded from analysis due to information in current literature about canine aging occurring significantly after the age of 8.

**Figure 1:**
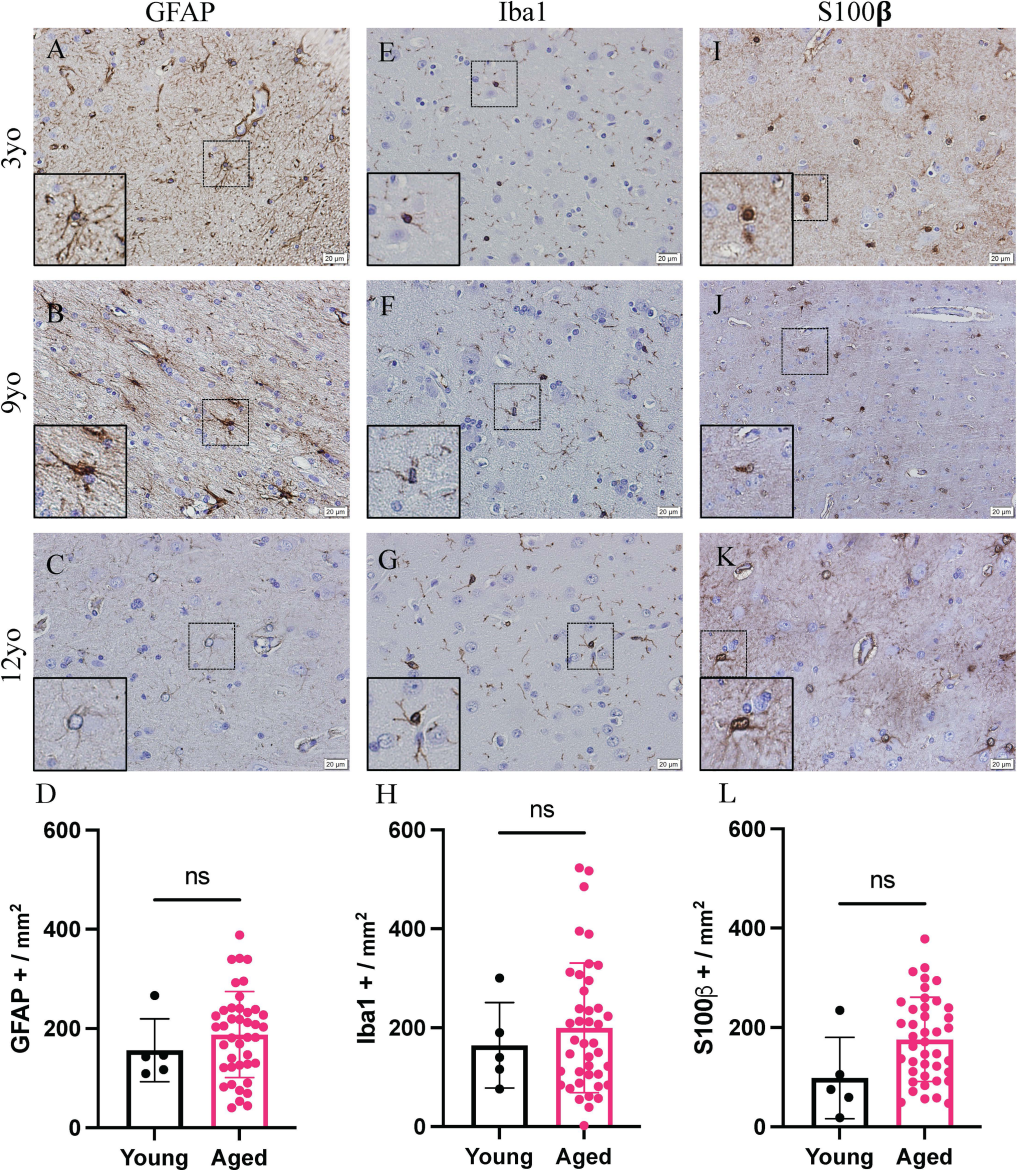
Increased glial reactivity in the cortex of aging canines. Increase no significant change was detected in GFAP+ cells **(A–D)**, IBA1+ cells **(E–H)** or S100β positive cells **(I–L)** compared to young canines. *n* = 5 young; *n* = 41 aged. Scale bar = 20 μM Unpaired *t*-test (**D**: *t* = 0.8882; **H**: *t* = 0.5822; **L**: *t* = 1.946. df = 44), error bars = SEM, *p* < 0.05. *p* values: *D* = 0.4348, *H* = 0.5634, L = 0.0580, ns = non-significance.

Representative images of the cortex of a 3-, 9- and 12-year-old canine stained for GFAP (Figures 1A–C), Iba1 (Figures 1E–G) and S100β (Figures 1I–K) is shown. No significant increase is seen in any of the glial cells, however a trending increase is present in all three. GFAP staining cells show a mean of 156.2 GFAP positive cells per mm^2^ in young canines compared to 187.8 GFAP positive cells per mm^2^ in aged canines (Figure 1D; *p* value = 0.4348; *t* = 0.7882; df = 44). In Iba1 staining, the mean for young canines is 164.4 Iba1 positive cells per mm^2^ while in aged canines it increases to 199.6 Iba1 positive cells per mm^2^ (Figure 1H; *p* value = 0.5634; *t* = 0.5822; df = 44). Finally, in S100β staining, the mean is 98.2 S100β positive cells per mm^2^ in young canines and 176.0 S100β positive cells per mm^2^ in aged canines (Figure 1L; *p* value = 0.0580; *t* = 1.946; df = 44).

**Figure 2:**
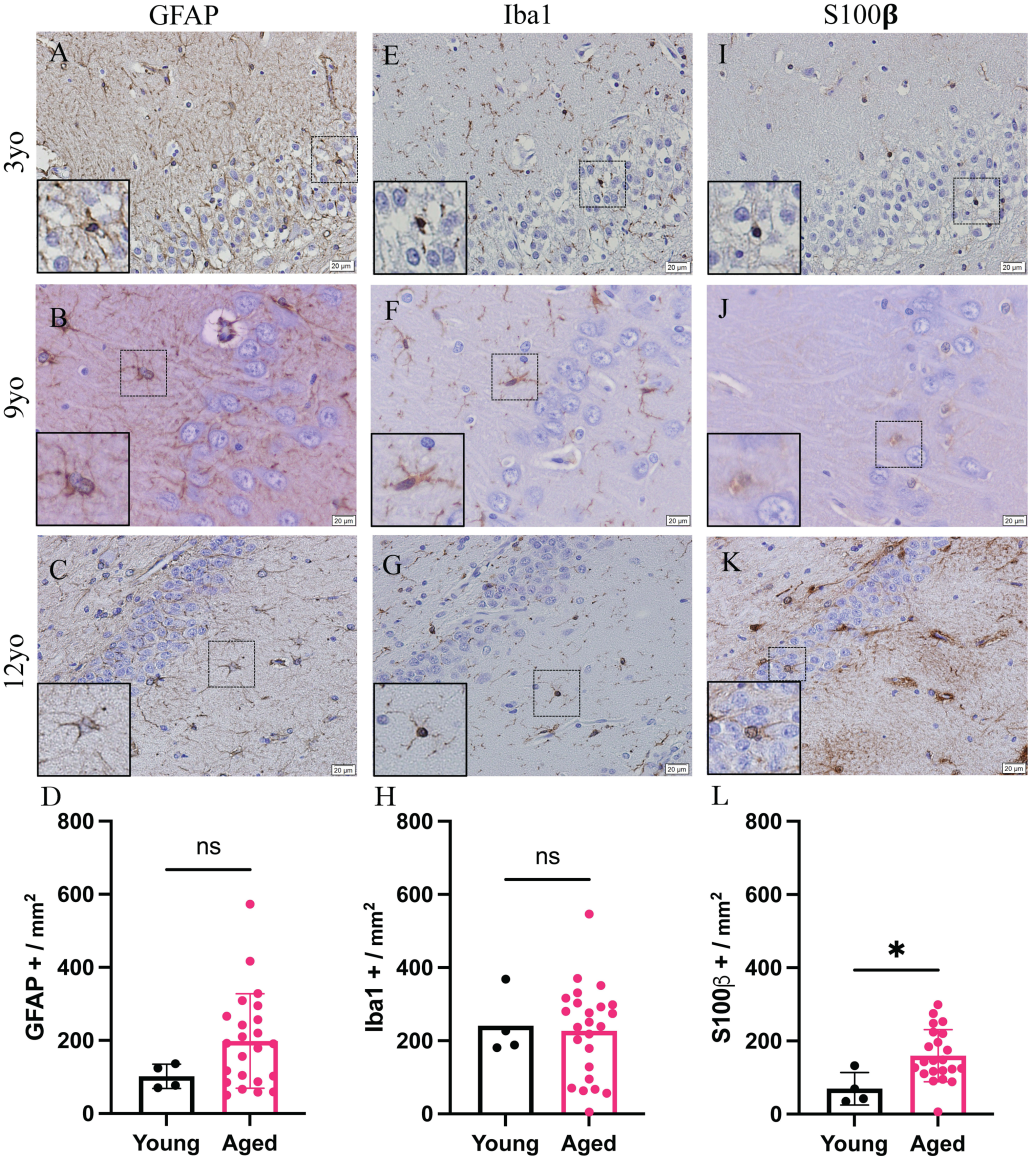
Increase glial reactivity in the hippocampus of aging canines. Increase in astrocyte reactivity detected by increase in S100β + (**J–L**; *n* = 25) in the hippocampus of aged canines when compared to young canines (**A,E,I**; *n* = 4). No significant change was detected in GFAP+ **(A–D)** or Iba1 + cells **(E–H)**. *n* = 4 young; *n* = 25 aged. Scale bar = 20 μM Unpaired *t*-test (**D**: *t* = 1.464; **H**: *t* = 0.2122; **L**: *t* = 2.416. df = 25), error bars = SEM, *p* < 0.05. *p* values: D = 0.1557, H = 0.8336, L = 0.0237 ns = non-significance.

All available brains with a hippocampus, 27 of the 46 dogs included in this study, were also analyzed. Due to the availability and nature of tissue received for dissection, not all canines had hippocampus present. Representative images of the hippocampus of 3-, 9- and 12-year-old canines stained for GFAP (Figures 2A–C), Iba1 (Figures 2E–G) and S100β (Figures 2I–K). A significant increase in S100β positive cells is also seen with a mean of 159.6 S100β positive cells per mm^2^ in aged canines compared to 69.5 S100β positive cells per mm^2^ in young canines (Figure 2L; *p* value = 0.0237; *t* = 1.464; df = 25), the difference means being 90.09 ± 37.29. No significant change, although a trend of an increase, was identified in the number of GFAP + and Iba1 + cells in the hippocampus (Figure 2D; *p* value = 0.1557; *t* = 0.2122; df = 25, Figure 2H; *p* value = 0.8336; *t* = 2.416; df = 25). Raw quantitative values of GFAP+, Iba1+, and S100b+ cells for each canine is listed in Table 2.

**Table 2:** Quantitative values of GFAP, Iba1 and S100beta for each canine in the coretex and hippocampus.

Canine ID	Cortex	GFAP + cells per mm^2^	Iba1 + cells per mm^2^	S100β + cells per mm^2^	Hippocampus	GFAP + cells per mm^2^	Iba1 + cells per mm^2^	S100β + cells per mm^2^
Ca110		109	76	234		77	188	69
Ca111		266	116	105		70	181	132
Ca112		146	190	59		124	227	42
Ca113		117	140	75		136	368	35
Ca152		241	517	133		N/A	N/A	N/A
Ca153		238	523	378		N/A	N/A	N/A
Ca154		339	485	199		220	251	253
Ca155		264	175	222		N/A	N/A	N/A
Ca156		143	300	18		N/A	N/A	N/A
Ca157		N/A	N/A	N/A		328	331	123
Ca159		227	209	236		N/A	N/A	N/A
Ca160		232	239	110		156	351	110
Ca163		169	312	111		58	546	148
Ca164		182	389	90		179	218	184
Ca169		218	150	162		242	274	146
Ca170		236	223	131		N/A	N/A	N/A
Ca171		140	326	171		309	237	95
Ca172		128	295	56		N/A	N/A	N/A
Ca173		126	168	92		66	239	117
Ca174		121	307	182		N/A	N/A	N/A
Ca175		53	329	147		102	280	119
Ca176		341	213	259		N/A	N/A	N/A
Ca178		388	207	126		N/A	N/A	N/A
Ca183		203	105	71		266	370	6
Ca184		167	61	294		N/A	N/A	N/A
Ca185		75	57	122		N/A	N/A	N/A
Ca186		203	84	49		N/A	N/A	N/A
Ca187		240	39	137		N/A	N/A	N/A
Ca188		90	169	83		573	95	N/A
Ca189		44	141	146		85	316	143
Ca192		147	395	102		257	298	248
Ca193		130	123	191		117	292	175
Ca194		122	212	219		N/A	N/A	N/A
Ca195		339	106	240		210	179	224
Ca196		292	274	299		N/A	N/A	N/A
Ca197		225	88	312		N/A	N/A	N/A
Ca198		295	238	251		N/A	N/A	275
Ca199		212	234	208		192	303	299
Ca250		205	81	47		N/A	N/A	N/A
Ca253		181	147	58		87	56	90
Ca256		40	111	206		104	70	125
Ca264		87	77	239		59	203	127
Ca289		182	2	253		417	67	624
Ca290		82	112	320		N/A	N/A	N/A
Ca294		69	84	92		191	63	196
Ca351		219	122	280		50	128	220
Ca800		208	55	190		295	5	88

N/A = data not available.

### 3.2. A1 Astrocytes increase in the cortex of aged canines

**Figure 3:**
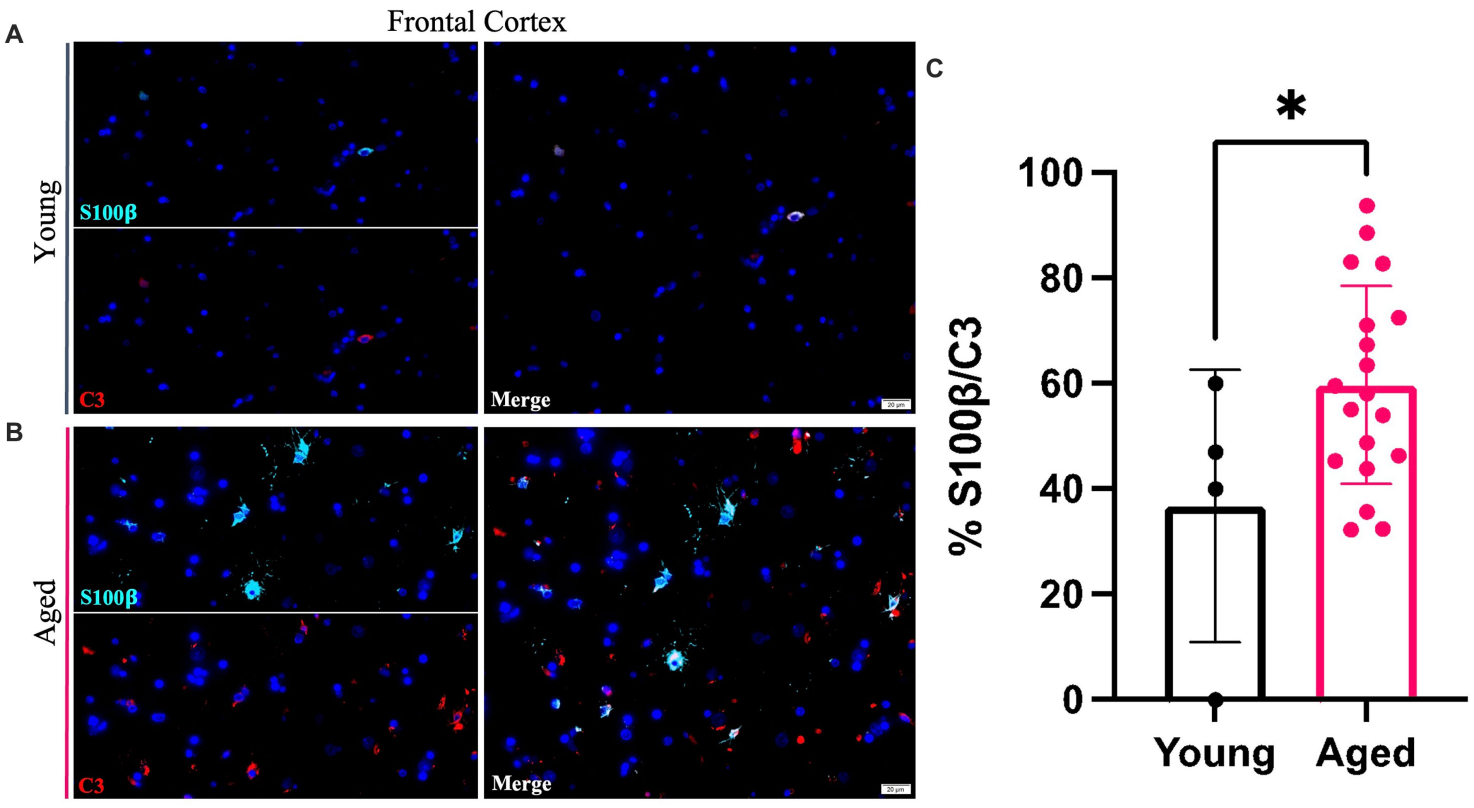
A1 reactive astrocytes increase within the cortex of the aging canine. An increase in C3 (red) co-localized with pan astrocyte marker S100b (green) is seen in aged canines **(B)** compared to young animals **(A)**. Quantification of these cells in the cortex reveals a significant increase in A1 astrocytes with age in this brain region **(C)**. (*n* = 22). Scale bar = 20 μM Unpaired *t*-test, *p* = 0.0486, error bars = SEM, *p* < 0.05.

Young and aged canine brains were analyzed to identify co-localization of S100β and C3 staining using co-immunofluorescence. Representative images show colocalization of S100β and C3 staining of the aged canine in the cortex (Figures 3A,B). A significant increase in the percent of S100β to C3 was found (Figure 3C), with *p* < 0.05 and the difference between means being 22.94 ± 10.95.

### 3.3. Phosphorylated tau and amyloid beta plaques increase in the cortex of aging canines

**Figure 4:**
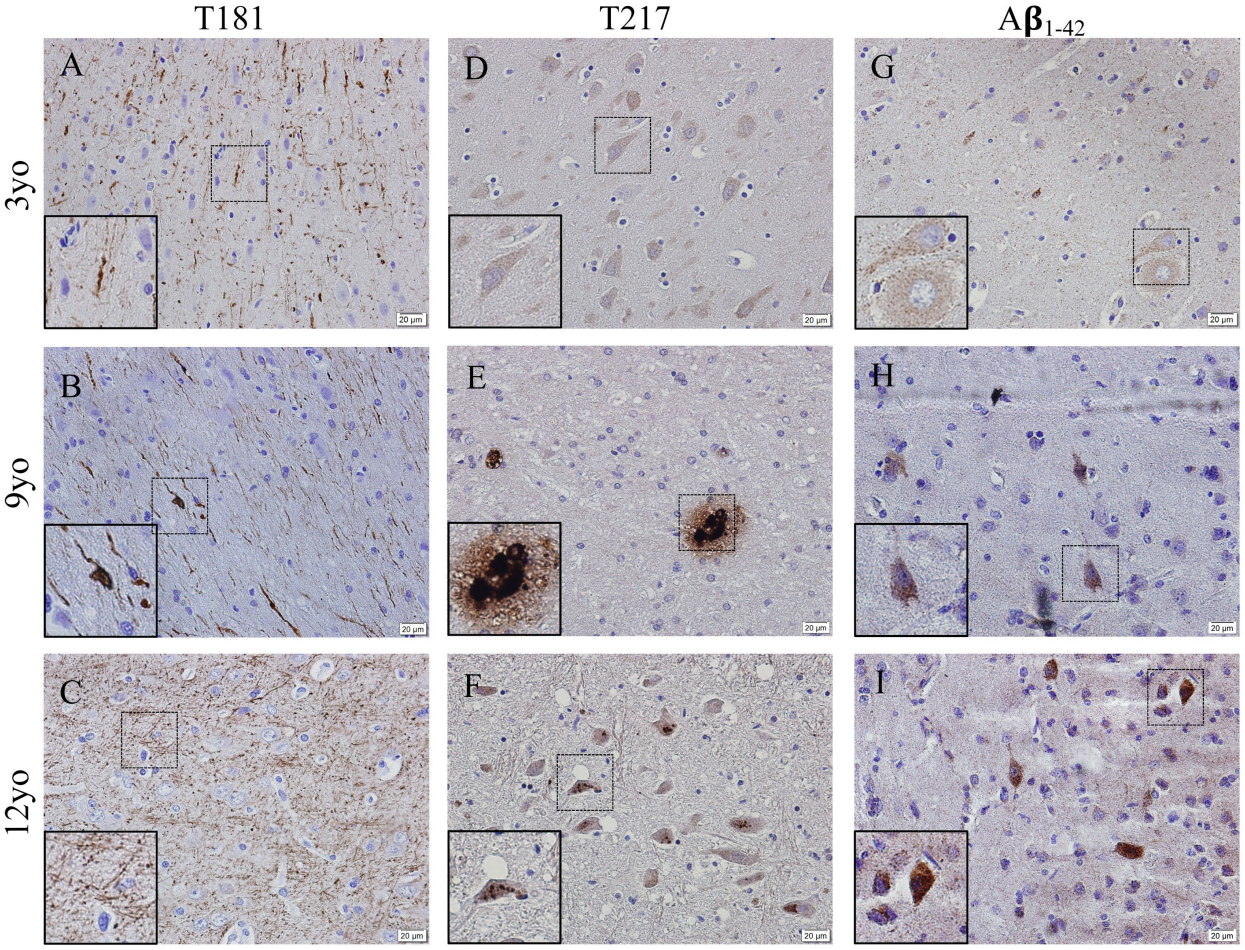
Hyperphosphorylation of Tau and Amyloid β_1-42_ accumulation in the cortex of aging canines. Representative images of young (3 years of age; *n* = 5) and aged (8 years of age and older; *n* = 41) canines are shown. Phosphorylated threonine 181 tau is increased at 9 **(B)** and 12 years of age **(C)** compared to young dog **(A)**. Phosphorylated threonine 217 tau is increased at 9 **(E)** and 12 years of age **(F)** with P-tau positive plaque like structure in (**E**, see inset) the 9-year-old dog and an intracellular phosphorylation in the 12-year-old canine (**F**, see inset) compared to young dog **(D)**. Aβ_1-42_ accumulates in the 9 **(H)** and 12 **(I)** year old dogs more than the young 3-year-old **(G)**. Scale bar = 20 μM.

Representative images of hyperphosphorylated tau and Aβ_1-42_ in the cortex from 3-, 9- and 12-year-old canines show possible deposits and tangles in the cortex (Figure 4). Two different phosphorylation sites of tau (P-tau) were analyzed by immunohistochemistry, including threonine 181 (P-tau-Thr181) (Figures 4A–C) and threonine 217 (P-tau-Thr217) (Figures 4D–F). An increase in intracellular accumulation and extracellular fibrils of P-tau is found in canines aged 9- and 12- years old compared to the young canine brain, aged 3 years. A possible formation of an extracellular neurofibrillary tangle with P-tau T217 antibody in the 9-year-old canine (see inset in Figure 4E) and intracellular P-tau aggregates in the 12-year-old canine (see inset Figure 4F) are identified. Intracellular amyloid-β_1-42_ aggregation was increased in the cortex of 9- and 12-year-old canines compared to young dogs, although no extracellular plaques are detected (Figures 4G–I).

### 3.4. Phosphorylated tau and amyloid beta plaques are present in the hippocampus of aging canines

**Figure 5:**
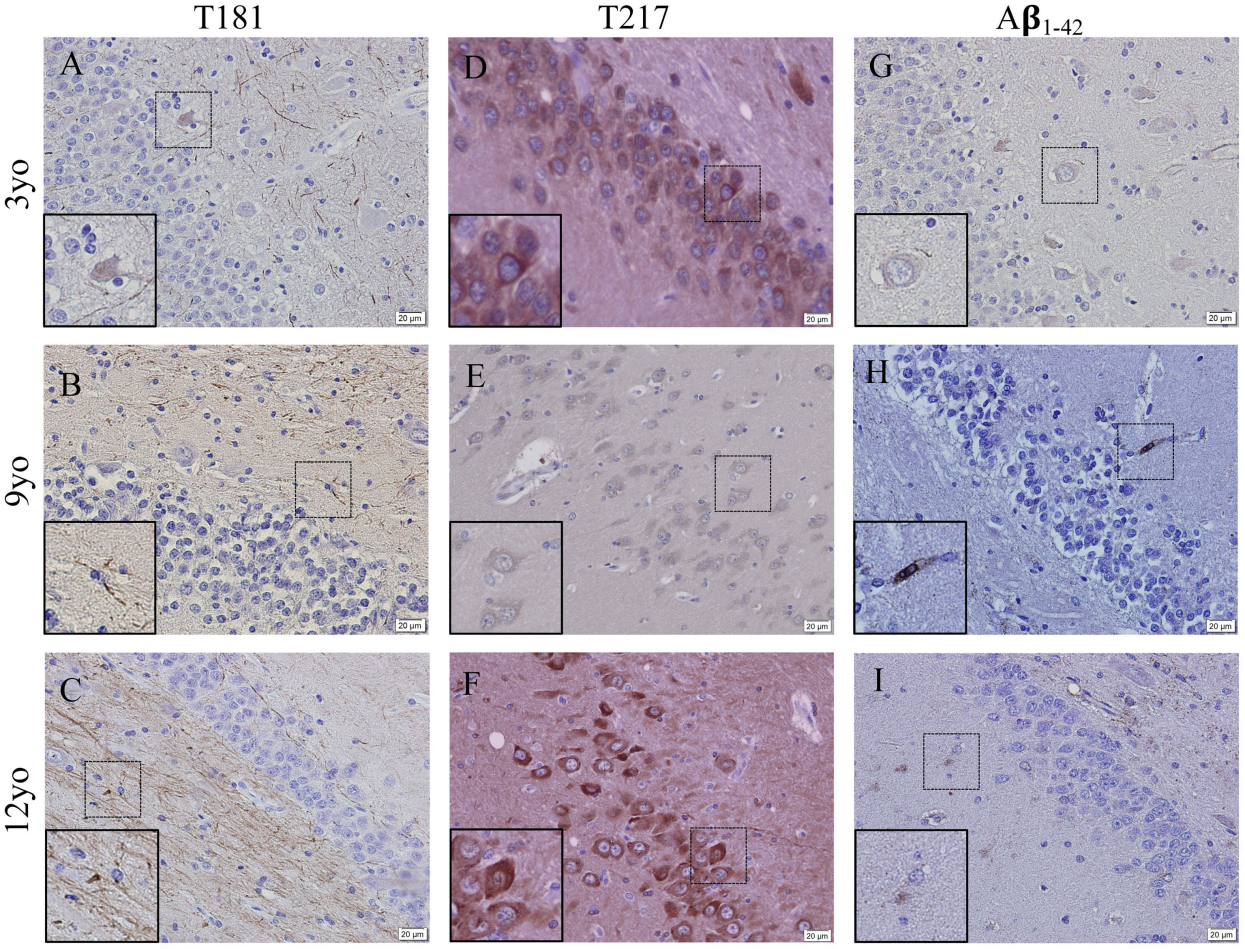
Hyperphosphorylation of Tau and Amyloid β_1-42_ accumulation in the hippocampus of young and aging canines. Representative images of young (3 years of age; *n* = 5) and aged (8 years of age and older; *n* = 41) canines are shown. Phosphorylated threonine 181 tau is increased at 9 **(B)** and 12 years of age **(C)** compared to young canines **(A)**. Phosphorylated threonine 217 tau is present at 3 years of age **(D)** and at 12 years of age with distinct intracellular deposits at 12 years of age **(F)** and very little staining at a different dog at 9 years old **(E)**. Aβ_1-42_ accumulates in the 9 **(H)** year old dog, more than the 3 year old **(G)** and 12 year old dog **(I)**. Scale bar = 20 μM.

Representative images of hyperphosphorylated tau and Aβ_1-42_ in the hippocampus of 3-, 9- and 12-year old canines possible tau hyperphosphorylation at two sites as well as amyloid beta_1-42_ aggregation. Possible tau fibrils are seen at tau phosphorylation site threonine 181 (Figures 5A–C) and at threonine 217 (Figures 5D–F), intracellular accumulation is seen in both 3- and 12-year old canines. Amyloid beta_1-42_ (Figures 5G–I) aggregation is seen (see inset in Figure 5H) in the 9-year old canine.

### 3.5. Questionnaires determined canine cognitive dysfunction in aging dogs and correlated to neuropathological results

**Figure 6:**
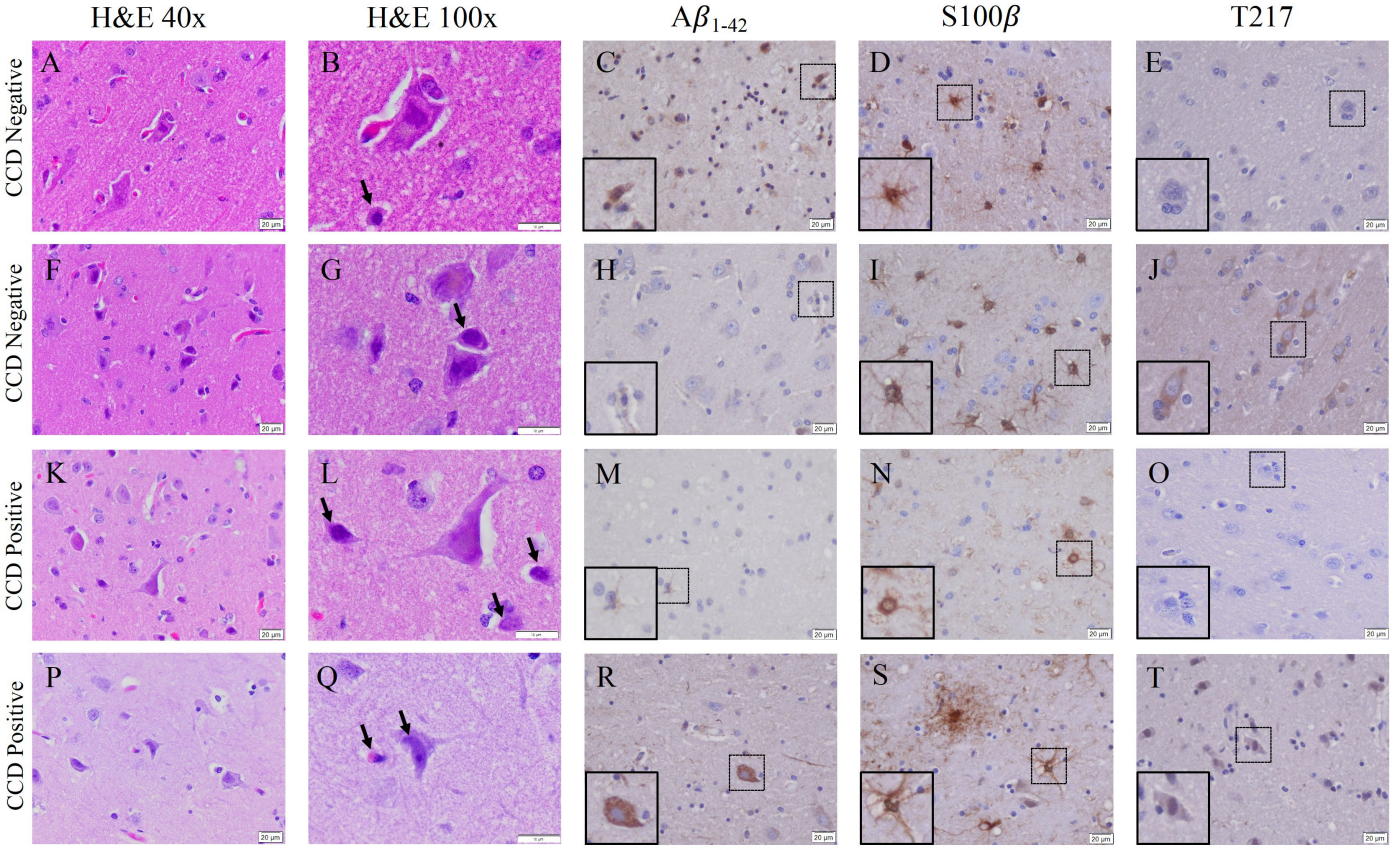
Gliosis and accumulation of misfolded proteins correlates to canines with positive cognitive dysfunction scores. **(A)** Aged canines with a CCDR/CADES score of being negative for CCD **(A–J)** does show an increase in gliosis and Aβ_1-42_ accumulation but no P-tau at T-217. **(B)** Aged canines with a moderate or severe CCD score **(K–T)** show gliosis and Aβ_1-42_ and P-tau positive cells. Canines negative or mild CADES *n* = 7; canines moderate or severe CADES *n* = 6. Scale bar = 20 μM.

The Canine Cognitive Dysfunction Rating Scale (CCDR) is based on a scale of 0–80 points, ranging from no abnormal behavior to severe behavioral disturbances (Salvin et al., 2011a). The CCDR has a diagnostic accuracy of 99.3% in dogs. In accordance with previously published data, we used a score of 50 for the CCDR questionnaire as an optimal diagnostic threshold, meaning it is expected that dogs scoring more than 50 points were likely to have CCD (Salvin et al., 2011a). The second questionnaire, Canine Dementia Scale (CADES), has been established as a validated screening tool for CCD, as well as a long-term assessment tool for progression and treatment efficacy (Madari et al., 2015). Dogs were classified as having either mild (1–7), moderate (scores of 24–44), or severe cognitive impairment (scores of 45–95). Of the 19 owner questionnaires we received from the aged canines, 26% were positive according to CCDR scoring and 32% of the dogs were classified as having severe cognitive impairment from the CADES questionnaire (Table 3). Another 26% of the dogs were classified as either mild or moderate for cognitive impairment using the CADES (Table 2). Critically, canines with either positive CCDR scores or severe impairment according to the CADES show an increase in both gliosis and accumulation of the misfolded proteins P-tau and Aβ_1-42_ compared to aged canines without CCD (Figure 6). We also through H&E staining were able to see an increase in pyknotic neurons within the aged brains with CCD positive surveys. However, some of the aged canines with a negative score for CCDR or CADES were not distinguishable from the CCD brains (Figures 6E–H). Canines with known comorbidities with the potential of impacting cognitive function were excluded from further analysis (Table 2).

**Table 3:** Scores of canines with CADES and CCDR owner questionnaires.

Canine ID	Age	CADES	Cognitive impairment	CCDR	CCD ±
Ca152	15yo	34	Moderate	49	Negative
Ca163	10yo	19	Mild	55	Positive
Ca169	8yo	0	None	n/a	N/A
Ca170	9yo	56	Severe	48	Negative
Ca173	14yo	4	None	36	Negative
Ca174	11yo	0	None	35	Negative
Ca183	14yo	0	None	34	Negative
Ca188	9yo	26	Moderate	34	Negative
Ca256	8yo	0	None	34	Negative
Ca264	13yo	0	None	35	Negative
Ca289	15yo	55	Severe	58	Positive
Ca351	13.6yo	49	Severe	51	Positive
Ca800	12.4yo	34	Moderate	45	Negative

## 4. Discussion

Aged dogs with CCD show promise for modeling human age-related neurodegeneration, including Alzheimer’s disease (AD) and other AD related dementias (ADRDs), and their respective hallmarks of disease, such as brain atrophy, cognitive decline, and Aβ in the CSF and brain (Pugliese et al., 2006; Yu et al., 2011; Schutt et al., 2015; Ozawa et al., 2016; Smolek et al., 2016). However, there is incomplete knowledge about how age-associated neuropathological progression relates to cognitive phenotypes in canines. This data is valuable from not only a veterinary perspective, but also a clinical one, as the canine is potentially a better model of age-related disease progression. The aged canine shares similar environmental factors to humans, unlike laboratory murine models, making them an ideal path to study the aging brain process. They also demonstrate critical elements of human disease, including neuroinflammation and aggregations of misfolded proteins. Here, we correlate gliosis to the accumulation of the neurotoxic proteins Aβ_1-42_ and P-tau in aged canines with and without CCD.

Microglia are essential for CNS homeostasis and play a role in age related neurodegenerative disease pathogenesis by contributing to an inflammatory brain state (Wang et al., 2015; Sobue et al., 2021). Our study is consistent with previous research that shows aged canines exhibit greater levels of the Iba1 protein in the hippocampus and canine cortical brain region compared to adult canines (Ozawa et al., 2016; Thomsen et al., 2021; Figures 1, 2). Similar to microglia, activated astrocytes produce numerous reactive and proinflammatory molecules in the AD brain (Heppner et al., 2015). Further, it is also known that microglial reactivity is followed by the progressive activation of astrocytes which are characterized by co-expression of C3 and S100β in AD human brains (Liddelow et al., 2017; Wu et al., 2019). In this study, we identify that C3 and S100β positive astrocytes are significantly increased in the cortex of aged canines. Additionally, quantifications of S100β^+^ cells show a significant increase in both the hippocampus and cortex. Although no significant increase of GFAP^+^ astrocytes is seen, these results are likely due to discrepancies in cellular expression of these proteins. Although more recent data has differentiated astrocytic populations into subtypes based on their transcriptional expression, data such as this is extremely limited in the canine. Murine studies show that there are populations of astrocytes, particularly in the hippocampus, that stain GFAP^−^. Considering the existence of this unique cell population that lacks the common glial marker, as well as evidence that S100β plays critical roles in astrocyte activation and migration, our data demonstrates the presence of gliosis in our canine model. Co-localization of C3 and S100 also establish the presence of reactive glial phenotypes in the canine brain, irrespective of their subtype (Figure 3).

Additionally, hippocampal and cortical astrocytes highly express genes related to glutamate receptor activity and synaptic transmission compared to populations in other brain regions (Lozzi et al., 2020). Reactive astrocytic phenotypes, such as those found in the cortex in aged canines, reduce glutamate uptake and subsequent conversion into its inactive form glutamine. Excessive accumulation of glutamate in the synaptic cleft overstimulates NMDA receptors, which are often upregulated in the aged brain to overcompensate for loss of function, especially in the cortex. Overstimulation eventually causes excitotoxicity of the neurons in those regions, contributing to cognition loss and overall neurodegeneration (Rothstein et al., 1996). Chronic activation of the glial cells in the hippocampus and cortex of CCD positive canines may cause dysregulation of glutamate uptake and synaptic transmission that exacerbates neuronal damage in addition to age-related mechanisms.

Two characteristic features of AD in humans are the presence of neurofibrillary tangles, composed of abnormally hyperphosphorylated tau protein, and Aβ plaques. Canines share tight AD gene sequence homology with humans (Sharman et al., 2013) and have alterations in amyloid precursor protein (APP) processing and tau immunoreactivity with age (Bates et al., 2014). Aβ deposits have been observed in aging dog brains, with the load of Aβ often correlated with CCD clinical signs (Head et al., 2010). The astrocyte marker S100β is also highly expressed by reactive astrocytes in close vicinity of beta amyloid deposits, and immunotherapies for Aβ in dogs reduces glial inflammation (Neus Bosch et al., 2015). Similarly, phosphorylated tau protein has been detected in the aging dog brain, although with less frequency and density than human AD brains (Smolek et al., 2016; Abey et al., 2021). Physiological tau is mostly confined to neuronal axons, where it is involved in microtubule stabilization. Microtubule binding, among other tau functions, are regulated by numerous serine, threonine, and tyrosine amino acid sites within the protein. Phosphorylation of these sites results in protein dysfunction and microtubule instability. Cytoplasmic deposits of tau phosphorylated at Threonine 181 have been previously recognized in the cortex of canines, but no neurofibrillary tangles (NFTs) have been identified (Smolek et al., 2016). In our study, we revealed fibril formation of tau phosphorylated at Thr181 and intracellular accumulation of tau phosphorylated at Thr217 in the cortices and hippocampus of aged canines compared to young animals (Figures 4, 5). Threonine 217 phosphorylation in the nine-year-old dog shows a unique, plaque-like structure or NFT. Although, to our knowledge, extracellular aggregation at this phosphorylation site has not been shown, this phosphorylation site is found in the proline-rich domain of the tau protein in close proximity to other amino acid residues commonly implicated in NFT formation (Xia et al., 2021). We were able to detect this finding in five of our aged canine brains. Different structures, conformations, and misfolds are associated with tauopathies, even within the same disease classification, which further highlights the variety on hyperphosphorylation and aggregation that may occur, especially in canines (Moloney et al., 2021; Wennstrom et al., 2022).

Lastly, it is critical that we are able to determine if these aging canines have CCD syndrome through the only current clinical diagnostic tool available, owner questionnaires (Salvin et al., 2010, 2011b; Ozawa et al., 2019; O'Brian et al., 2021). To determine this, we asked owners to complete both the CCDR and CADES questionnaires on necropsied dogs, as none were diagnosed with CCD at the time of death (Table 1). All owners of the canines we have performed pathological analysis on were contacted and 19, or 40%, of the surveys were returned for analysis (Table 2). However, pathology of the dogs with either severe criteria from the CADES questionnaire or CCDR positive for dementia show pathologies that were not always distinguishable from age matched canine brains. We found no significant change in the accumulation and aggregation of P-tau and Aβ (Figures 6N,P) in canines with CCD compared to aged, matched canines without CCD in all of our canine brains. Although misfolded protein aggregates are characteristic of neurodegenerative disease, their presence alone does not equate to declined cognitive state and neuropathological damage. Aggregates themselves do not cause neuronal death, rather, they contribute to neuronal dysfunction and exacerbate damaging, chronic neuroinflammatory signaling. The presence of misfolded proteins in correlation with increased numbers of phenotypically neurotoxic glia, as seen in the CCD positive animals, may intensify neuronal damage and loss. This is seen by an increased number of punctate neurons (Figure 6M), indicative of apoptotic or dying neurons, identified in aged canines with CCD compared to aged canines without CCD. Together, these data address the potential cause of cognition loss associated with CCD outside of normal aging.

### 4.1. Study limitations

The variability of our data indicates that there are a few limitations to our study that could explain variability within our findings. The small sample size of owner questionnaires that we received may not be entirely accurate, as these were performed after their dog had passed, and in some cases even years afterword. We were also unable to perform a medical review to rule out other causes of clinical disease, such as structural abnormalities in the brain or infection. Therefore, we are unsure if the absence of significance is due to the lack of power for the clinical questionaries, the lack of a full medical review, or that there is no true difference between aspects of aging pathology and CCD clinical diagnosis. The tissues analyzed was also a limitation of this study. We were only able to use available necropsied tissue, which meant that not all canine brain sample had the hippocampus brain regions. Also due to our small sample size we are unable to assess the role of breed differences in aging currently. We therefore chose to use eight years of age as our cutoff irrespective of the breed to determine in general the neuropathological changes induced. We are award this is an extreme limitation of our study and hope with time we will be able to expand these findings to better understand breed differences. Future studies that are well powered, include a complete medical review (to rule out other medical conditions that could cause the neuropathology) and have timely owner questionnaires are necessary for future development of this research.

### 4.2. Conclusion

The aging brain and the factors that lead to susceptibility to diseases like cognitive decline is still unknown. To better understand this, we have studied thirty-eight naturally aged canine brains for pathological markers of glial inflammation, morphological changes, accumulation of amyloid β_1-42_ and hyperphosphorylation of tau. Both microglial and astrocyte number are increased, making this the first report to our knowledge detecting activation of reactive astrocytes defined by co staining with complement 3 (C3) and S100β in naturally aging canines, a similar phenotype found in Alzheimer’s disease laboratory rodent models and patients. We also detect Aβ_1-42_ accumulation and T181 and T217 for hyperphosphorylation of tau in most of the aged brain samples in this cohort. Following owner questionnaires, 19 of these aged dogs, 26% were positive for CCD based on the CCDR; however, there was no correlation between these biomarkers and CCD. However, future studies with timely clinical medical review and questionaries may allow us to determine differences between the two aging populations.

## Data availability statement

The raw data supporting the conclusions of this article will be made available by the authors, without undue reservation.

## Ethics statement

The animal study was reviewed and approved by College of Veterinary and Biomedical Sciences College Regulatory Broad (CRB) and the Colorado State University IACUC committee. Written informed consent was obtained from the owners for the participation of their animals in this study.

## Author contributions

JM and SM conceived and designed the project. AH, AL, and MR performed the pathology experiments. BK and LM collected canine samples and owner questionnaires. AH, AL, SM, and JM contributed to data analysis. JM, AH and AL wrote the manuscript. All authors reviewed and edited the manuscript.

## Funding

This research received funding from the Colorado State University Translational Medicine Institute and Office of Vice President of Research Catalyst Innovative Program- for the Center for Healthy Aging.

## Conflict of interest

The authors declare that the research was conducted in the absence of any commercial or financial relationships that could be construed as a potential conflict of interest.

## Publisher’s note

All claims expressed in this article are solely those of the authors and do not necessarily represent those of their affiliated organizations, or those of the publisher, the editors and the reviewers. Any product that may be evaluated in this article, or claim that may be made by its manufacturer, is not guaranteed or endorsed by the publisher.

## References

[ref1] Abey A. Davies D. Goldsbury C. Buckland M. Valenzuela M. Duncan T. (2021). Distribution of tau hyperphosphorylation in canine dementia resembles early Alzheimer's disease and other tauopathies. Brain Pathol. 31, 144–162. doi: 10.1111/bpa.12893, PMID: 32810333 PMC8018065

[ref2] Azkona G. Garcia-Belenguer S. Chacon G. Rosado B. Leon M. Palacio J. (2009). Prevalence and risk factors of behavioural changes associated with age-related cognitive impairment in geriatric dogs. J. Small Anim. Pract. 50, 87–91. doi: 10.1111/j.1748-5827.2008.00718.x, PMID: 19200264

[ref3] Barnes J. Cotton P. Robinson S. Jacobsen M. (2016). Spontaneous pathology and routine clinical pathology parameters in aging beagle dogs: a comparison with adolescent and young adults. Vet. Pathol. 53, 447–455. doi: 10.1177/0300985815610390, PMID: 26553522

[ref4] Bates K. Vink R. Martins R. Harvey A. (2014). Aging, cortical injury and Alzheimer's disease-like pathology in the Guinea pig brain. Neurobiol. Aging 35, 1345–1351. doi: 10.1016/j.neurobiolaging.2013.11.020, PMID: 24360504

[ref5] Beach T. G. Walker R. McGeer E. G. (1989). Patterns of gliosis in Alzheimer's disease and aging cerebrum. Glia 2, 420–436. doi: 10.1002/glia.440020605 2531723

[ref6] Cummings B. J. Cotman C. W. (1995). Image analysis of beta-amyloid load in Alzheimer's disease and relation to dementia severity. Lancet 346, 1524–1528. doi: 10.1016/S0140-6736(95)92053-6, PMID: 7491048

[ref7] Head E. Callahan H. Muggenburg B. A. Cotman C. W. Milgram N. W. (1998). Visual-discrimination learning ability and beta-amyloid accumulation in the dog. Neurobiol. Aging 19, 415–425. doi: 10.1016/S0197-4580(98)00084-0, PMID: 9880044

[ref8] Head E. Pop V. Sarsoza F. Kayed R. Beckett T. L. Studzinski C. M.. (2010). Amyloid-beta peptide and oligomers in the brain and cerebrospinal fluid of aged canines. J. Alzheimers Dis. 20, 637–646. doi: 10.3233/JAD-2010-1397, PMID: 20164551 PMC2903832

[ref9] Heppner F. L. Ransohoff R. M. Becher B. (2015). Immune attack: the role of inflammation in Alzheimer disease. Nat. Rev. Neurosci. 16, 358–372. doi: 10.1038/nrn3880 25991443

[ref10] Hwang I. K. Lee C. H. Li H. Yoo K. Y. Choi J. H. Kim D. W.. (2008). Comparison of ionized calcium-binding adapter molecule 1 immunoreactivity of the hippocampal dentate gyrus and CA1 region in adult and aged dogs. Neurochem. Res. 33, 1309–1315. doi: 10.1007/s11064-007-9584-6, PMID: 18270819

[ref11] Johnstone E. M. Chaney M. O. Norris F. H. Pascual R. Little S. P. (1991). Conservation of the sequence of the Alzheimer's disease amyloid peptide in dog, polar bear and five other mammals by cross-species polymerase chain reaction analysis. Brain Res. Mol. Brain Res. 10, 299–305. doi: 10.1016/0169-328X(91)90088-F, PMID: 1656157

[ref12] Liddelow S. A. Guttenplan K. A. Clarke L. E. Bennett F. C. Bohlen C. J. Schirmer L.. (2017). Neurotoxic reactive astrocytes are induced by activated microglia. Nature 541, 481–487. doi: 10.1038/nature21029, PMID: 28099414 PMC5404890

[ref13] Lozzi B. Huang T.-W. Sardar D. Huang A. Y.-S. Deneen B. (2020). Regionally distinct astrocytes display unique transcription factor profiles in the adult Brain. Front. Neurosci. 14:61. doi: 10.3389/fnins.2020.00061, PMID: 32153350 PMC7046629

[ref14] Madari A. Farbakova J. Katina S. Smolek T. Novak P. Weissova T.. (2015). Assessment of severity and progression of canine cognitive dysfunction syndrome using the CAnine DEmentia scale (CADES). Appl. Anim. Behav. Sci. 171, 138–145. doi: 10.1016/j.applanim.2015.08.034

[ref15] Moloney C. M. Lowe V. J. Murray M. E. (2021). Visualization of neurofibrillary tangle maturity in Alzheimer's disease: a clinicopathologic perspective for biomarker research. Alzheimers Dement. 17, 1554–1574. doi: 10.1002/alz.12321, PMID: 33797838 PMC8478697

[ref16] Mormino E. C. Papp K. V. (2018). Amyloid accumulation and cognitive decline in clinically Normal older individuals: implications for aging and early Alzheimer's disease. J. Alzheimers Dis. 64, S633–S646. doi: 10.3233/JAD-179928, PMID: 29782318 PMC6387885

[ref17] Neilson J. C. Hart B. L. Cliff K. D. Ruehl W. W. (2001). Prevalence of behavioral changes associated with age-related cognitive impairment in dogs. J. Am. Vet. Med. Assoc. 218, 1787–1791. doi: 10.2460/javma.2001.218.1787, PMID: 11394831

[ref18] Neus Bosch M. Pugliese M. Andrade C. Gimeno-Bayon J. Mahy N. Rodriguez M. J. (2015). Amyloid-beta immunotherapy reduces amyloid plaques and astroglial reaction in aged domestic dogs. Neurodegener Dis. 15, 24–37. doi: 10.1159/000368672, PMID: 25531153

[ref19] O'Brian M. L. Herron M. E. Smith A. M. Aarnes T. K. (2021). Effects of a four-week group class created for dogs at least eight years of age on the development and progression of signs of cognitive dysfunction syndrome. J. Am. Vet. Med. Assoc. 259, 637–643. doi: 10.2460/javma.259.6.637, PMID: 34448609

[ref20] Ozawa M. Chambers J. K. Uchida K. Nakayama H. (2016). The relation between canine cognitive dysfunction and age-related brain lesions. J. Vet. Med. Sci. 78, 997–1006. doi: 10.1292/jvms.15-0624, PMID: 26922972 PMC4937160

[ref21] Ozawa M. Inoue M. Uchida K. Chambers J. K. Takeuch Y. Nakayama H. (2019). Physical signs of canine cognitive dysfunction. J. Vet. Med. Sci. 81, 1829–1834. doi: 10.1292/jvms.19-0458, PMID: 31685716 PMC6943310

[ref22] Pekna M. Pekny M. (2021). The complement system: a powerful modulator and effector of astrocyte function in the healthy and diseased central nervous system. Cells 10:1812. doi: 10.3390/cells10071812, PMID: 34359981 PMC8303424

[ref23] Pugliese M. Carrasco J. L. Andrade C. Mas E. Mascort J. Mahy N. (2005). Severe cognitive impairment correlates with higher cerebrospinal fluid levels of lactate and pyruvate in a canine model of senile dementia. Prog. Neuro Psychopharmacol. Biol. Psychiatry 29, 603–610. doi: 10.1016/j.pnpbp.2005.01.017, PMID: 15866364

[ref24] Pugliese M. Geloso M. C. Carrasco J. L. Mascort J. Michetti F. Mahy N. (2006). Canine cognitive deficit correlates with diffuse plaque maturation and S100beta (−) astrocytosis but not with insulin cerebrospinal fluid level. Acta Neuropathol. 111, 519–528. doi: 10.1007/s00401-006-0052-1, PMID: 16718348

[ref25] Rofina J. E. van Ederen A. M. Toussaint M. J. Secreve M. van der Spek A. van der Meer I.. (2006). Cognitive disturbances in old dogs suffering from the canine counterpart of Alzheimer's disease. Brain Res. 1069, 216–226. doi: 10.1016/j.brainres.2005.11.021, PMID: 16423332

[ref26] Rothstein J. D. Dykes-Hoberg M. Pardo C. A. Bristol L. A. Jin L. Kuncl R. W.. (1996). Knockout of glutamate transporters reveals a major role for Astroglial transport in Excitotoxicity and clearance of glutamate. Neuron 16, 675–686. doi: 10.1016/S0896-6273(00)80086-0, PMID: 8785064

[ref27] Salvin H. E. McGreevy P. D. Sachdev P. S. Valenzuela M. J. (2010). Under diagnosis of canine cognitive dysfunction: a cross-sectional survey of older companion dogs. Vet. J. 184, 277–281. doi: 10.1016/j.tvjl.2009.11.007, PMID: 20005753

[ref28] Salvin H. E. McGreevy P. D. Sachdev P. S. Valenzuela M. J. (2011a). The canine cognitive dysfunction rating scale (CCDR): a data-driven and ecologically relevant assessment tool. Vet. J. 188, 331–336. doi: 10.1016/j.tvjl.2010.05.014, PMID: 20542455

[ref29] Salvin H. E. McGreevy P. D. Sachdev P. S. Valenzuela M. J. (2011b). The canine sand maze: an appetitive spatial memory paradigm sensitive to age-related change in dogs. J. Exp. Anal. Behav. 95, 109–118. doi: 10.1901/jeab.2011.95-109, PMID: 21541168 PMC3014775

[ref30] Schutt T. Toft N. Berendt M. (2015). Cognitive function, progression of age-related behavioral changes, biomarkers, and survival in dogs more than 8 years old. J. Vet. Intern. Med. 29, 1569–1577. doi: 10.1111/jvim.13633, PMID: 26463980 PMC4895687

[ref31] Sharman M. J. Moussavi Nik S. H. Chen M. M. Ong D. Wijaya L. Laws S. M.. (2013). The Guinea pig as a model for sporadic Alzheimer's disease (AD): the impact of cholesterol intake on expression of AD-related genes. PLoS One 8:e66235. doi: 10.1371/journal.pone.0066235, PMID: 23805206 PMC3689723

[ref32] Smolek T. Madari A. Farbakova J. Kandrac O. Jadhav S. Cente M.. (2016). Tau hyperphosphorylation in synaptosomes and neuroinflammation are associated with canine cognitive impairment. J. Comp. Neurol. 524, 874–895. doi: 10.1002/cne.23877, PMID: 26239295

[ref33] Sobue A. Komine O. Hara Y. Endo F. Mizoguchi H. Watanabe S.. (2021). Microglial gene signature reveals loss of homeostatic microglia associated with neurodegeneration of Alzheimer's disease. Acta Neuropathol. Commun. 9:1. doi: 10.1186/s40478-020-01099-x, PMID: 33402227 PMC7786928

[ref34] Thomsen B. B. Madsen C. Krohn K. T. Thygesen C. Schutt T. Metaxas A.. (2021). Mild microglial responses in the cortex and perivascular macrophage infiltration in subcortical white matter in dogs with age-related dementia Modelling prodromal Alzheimer's disease. J. Alzheimers Dis. 82, 575–592. doi: 10.3233/JAD-210040, PMID: 34057083 PMC8385501

[ref35] Wang W. Y. Tan M. S. Yu J. T. Tan L. (2015). Role of pro-inflammatory cytokines released from microglia in Alzheimer's disease. Ann. Transl. Med. 3:136. doi: 10.3978/j.issn.2305-5839.2015.03.49 26207229 PMC4486922

[ref36] Wennstrom M. Janelidze S. Nilsson K. P. R. Netherlands Brain B. Serrano G. E. Beach T. G.. (2022). Cellular localization of p-tau217 in brain and its association with p-tau217 plasma levels. Acta Neuropathol. Commun. 10:3. doi: 10.1186/s40478-021-01307-2, PMID: 34991721 PMC8734209

[ref37] Wu T. Dejanovic B. Gandham V. D. Gogineni A. Edmonds R. Schauer S.. (2019). Complement C3 is activated in human AD Brain and is required for Neurodegeneration in mouse models of amyloidosis and Tauopathy. Cell Rep. 28, 2111–2123.e6. doi: 10.1016/j.celrep.2019.07.060, PMID: 31433986

[ref38] Xia Y. Prokop S. Giasson B. I. (2021). “Don’t Phos over tau”: recent developments in clinical biomarkers and therapies targeting tau phosphorylation in Alzheimer’s disease and other tauopathies. Mol. Neurodegener. 16:37. doi: 10.1186/s13024-021-00460-5, PMID: 34090488 PMC8180161

[ref39] Yu C. H. Song G. S. Yhee J. Y. Kim J. H. Im K. S. Nho W. G.. (2011). Histopathological and immunohistochemical comparison of the brain of human patients with Alzheimer's disease and the brain of aged dogs with cognitive dysfunction. J. Comp. Pathol. 145, 45–58. doi: 10.1016/j.jcpa.2010.11.004, PMID: 21256508

